# Supplementary material for: The causal relationship between the human gut microbiota and pyogenic arthritis: a Mendelian randomization study
Source: Front Cell Infect Microbiol. 2024 Nov 26;14:1452480. doi: 10.3389/fcimb.2024.1452480 (PMC11629706; doi:10.3389/fcimb.2024.1452480)
Supplement: Supplementary file 1 [file DataSheet1.zip › mendelian test/ebi-a-GCST90027529.csv_forest.pdf]

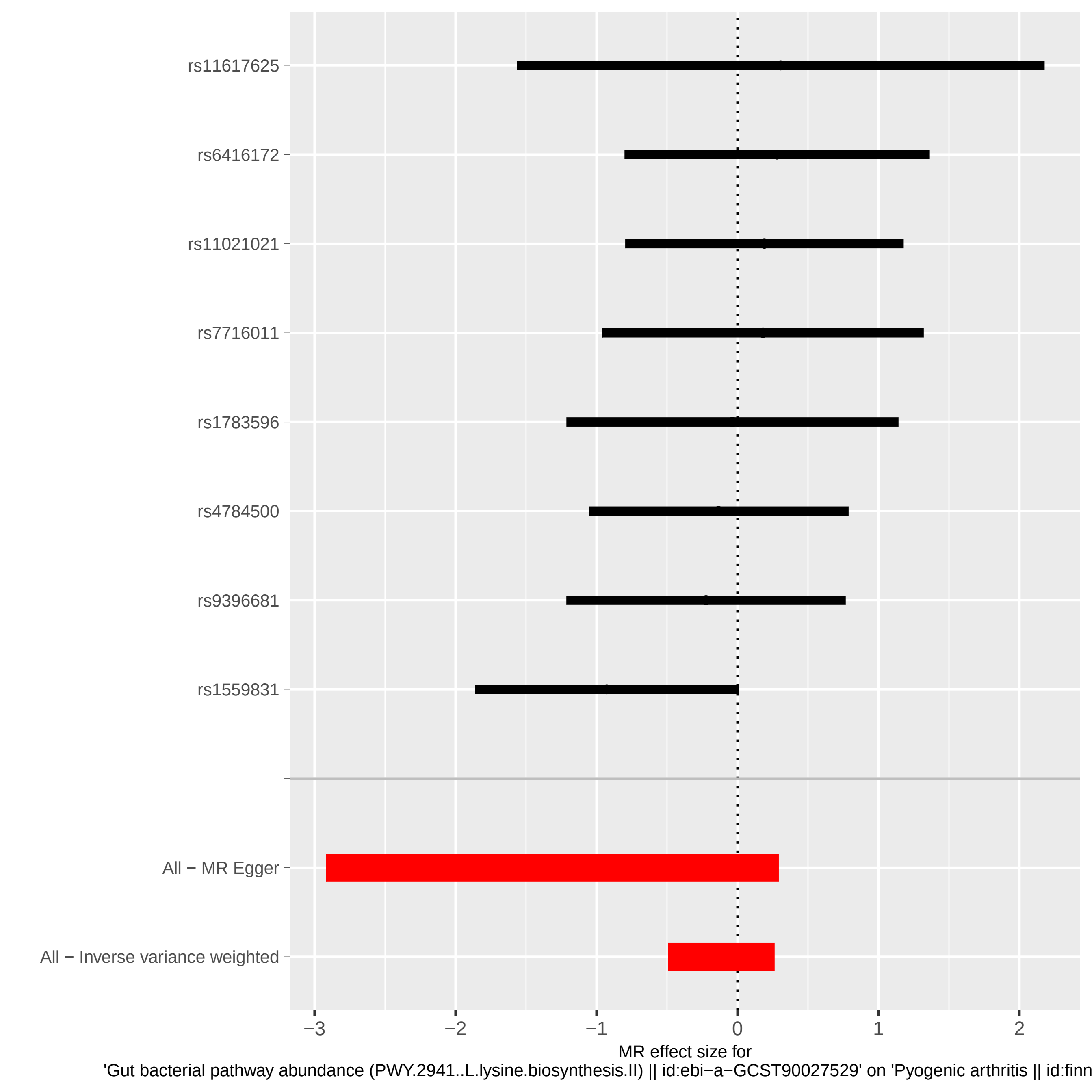
rs11617625
rs6416172
rs11021021
rs7716011
rs1783596
rs4784500
rs9396681
rs1559831
All − MR Egger
All − Inverse variance weighted
−3
−2
−1
0
1
2
MR effect size for
'Gut bacterial pathway abundance (PWY.2941..L.lysine.biosynthesis.II) || id:ebi−a−GCST90027529' on 'Pyogenic arthritis || id:finn
